# Allergic contact dermatitis to cosmetics: retrospective analysis of a population subjected to patch tests between 2004 and 2017^⋆^^⋆⋆^

**DOI:** 10.1016/j.abd.2020.04.011

**Published:** 2020-09-17

**Authors:** Mariana de Figueiredo Silva Hafner, Ana Carolina Rodrigues, Rosana Lazzarini

**Affiliations:** a Dermatology Clinic, Santa Casa de Misericórdia de São Paulo, São Paulo, SP, Brazil; b Medical Sciences College, Santa Casa de Misericórdia de São Paulo, São Paulo, SP, Brazil

**Keywords:** Cosmetics, Dermatitis, allergic contact, Patch tests

## Abstract

**Background:**

Cosmetics are part of the daily life of the population, and their use can lead to allergic contact dermatitis.

**Objectives:**

To assess the profile of patients diagnosed with allergic contact dermatitis to cosmetics treated at a referral center for 13 years, as well as the characteristics of the clinical picture and allergens involved.

**Methods:**

This was a retrospective study, with analysis of medical records of patients attended at this service. The individuals included had a diagnostic hypothesis of allergic contact dermatitis to cosmetics and had previously been submitted to epicutaneous tests.

**Results:**

A total of 1405 medical records were analyzed, 403 (28.7%) with suspected allergic contact dermatitis to cosmetics and 232 (16.5%) with confirmed diagnosis. Of these, 208 (89.7%) were women, and the age group most affected was 31 − 60 years. The most common locations were face in 195 cases (25.8%), cervical region in 116 (15.3%), and trunk in 96 (12.6%). The main allergens in the contact tests were toluene-sulfonamide-formaldehyde resin in 69 cases (29.7%), paraphenylenediamine in 54 (26.3%), Kathon CG® in 41 (20.7%), and fragrance-mix 1 in 29 (16.4%). In 154 (66.4%) of the 232 patients with a confirmed diagnosis of allergic contact dermatitis to cosmetics it was possible to specify the cosmetic product responsible for the lesions.

**Study limitations:**

The absence of some allergens considered important in the world as causes of allergic contact dermatitis, which are not readily accessible among us.

**Conclusions:**

The data of the analyzed population (predominance of young women), as well as the location of the lesions (face and cervical area) and the main allergens involved were consistent with those from the world literature.

## Introduction

The term ‘cosmetics’ is defined by the Brazilian National Health Surveillance Agency (Agência Nacional de Vigilância Sanitária [ANVISA]) as products for external use, intended to protect or beautify different parts of the body. The Food and Drug Administration (FDA) defines them as articles that can be applied to the human body for cleaning, beautifying, highlighting features, changing appearance, or even as components of any of these products, with the exception of soaps.^1^ In accordance with the European legislation, the term is used for a substance or mixture of substances intended for application on the external surfaces of the body (skin, hair, nails, lips, and genitalia), teeth and/or oral mucosa, with the purpose of cleaning, odorization, modification of appearance, or correction of odors in the region of use.^2^ Thus, this term varies according to the legislation of each country and includes makeup, skin care items, perfumes, hair and nail products, shaving gels or creams, and any personal care products, such as toothpaste and deodorants.^3^ Cosmetics are part of the daily life of the population, being more used by women, who, on average, apply about 12 products per day, which can contain up to 168 different components, while men use up to six products with an average of 85 components.^4^

Although the most common adverse effects caused by the use of cosmetics are irritant dermatitis, allergic contact dermatitis (ACD) also occurs, corresponding to about 1% of the reactions. The incidence of ACD varies according to the region, frequency of use of cosmetics, allergenic power of the products used, and access to patch tests (which confirm the diagnosis). The risk factor for its occurrence is the increase in the use of cosmetics; thus, the population most affected is females between 20 and 55 years of age. It is difficult to estimate the frequency of this condition, since most individuals do not seek medical services when experiencing such reactions and discontinue the use on their own.^1^, ^3^

Hygiene products and moisturizers are the main responsible for the cases of ACD to cosmetics, followed by makeup, hair products, and nail products.^2^ The main associated allergens are fragrances and preservatives.^4^

ACD to cosmetics occurs at the place of direct application of the product or to which it can be transferred. This transmission can occur through unintentional contact through objects such as towels and phones, through air, and through interpersonal contact.^1^ The clinical picture can be acute or chronic; chronic manifestations are the most common, due to the low allergenic power of the cosmetic components.^3^

The diagnosis is based on anamnesis, dermatological examination, and patch test. It is important to investigate the products used by the patient, both at home and at work. After diagnosis, it is essential that the patient understands that she/he should avoid contact with the allergen and that the condition may return with new exposures.^1^, ^3^, ^4^

The present study aimed to assess the profile of patients diagnosed with ACD to cosmetics treated at a referral center for a period of 13 years, as well as the characteristics of the clinical picture presented and the allergens involved.

## Materials and methods

This was a retrospective study, with analysis of the medical records of patients treated at a reference service outpatient clinic during the period from 2004 to 2017. It was approved by the Human Research Ethics Committee (CAAE 94354218.4.0000.5479).

The patients included in the study had a diagnostic hypothesis of ACD to cosmetics and had previously been submitted to epicutaneous tests. According to the anamnesis, the series of allergens used in the tests were: Brazilian standard panel (30 substances/FDA – Allergenic/RJ, Brazil), cosmetics panel (ten substances/FDA – Allergenic, RJ, Brazil), and when possible, cosmetic products that belonged to the patient herself/himself was also included. In all cases, the containers used in the tests were of the Finn Chamber (Smart Practice, United States) or Allergo Chamber (Neoflex, São Paulo, Brazil) type. The tests were applied and read according to the criteria of the International Contact Dermatitis Research Group (ICDRG).

In order to standardize the methodology and the reading of the patch tests, avoiding the occurrence of false positives and false negatives, the ICDRG established that the tests must be applied on the high portion of the back of the patients, and the readings must be taken after 48 and 96 h. The reading can show negative or positive results (1, 2, or 3 +), as shown in table 1.

**Table 1:** Reading of the results of patch tests (ICDRG criteria).

Intensity	Morphology	Interpretation
–	No reaction	Negative
?+	Mild erythema	Doubtful reaction
+	Erythema, infiltration, possibly papules	Poor response
++	Erythema, infiltration, papules, vesicles	Strong response
+++	Intense erythema, infiltration, coalescent vesicles	Very strong response

Data were collected according to a standard form adopted by the service, in which information such as age, sex, profession, duration of the condition, location of lesions, and results of patch tests are recorded. The collected data was analyzed in an Excel spreadsheet.

## Results

A total of 1405 medical records were analyzed, among which 403 (28.7%) were suspected of having ACD due to cosmetics and 232 (16.5%) had this diagnosis confirmed. In this group, 208 (89.7%) were female and 24 (10.3%) were male. The referred history period varied from 1 to 528 months, with a mean duration of 32.9 months.

The patients' ages ranged from 3 to 88 years; the age group most affected was from 31 − 60 years, with a mean age of 44.4 years.

The most common locations were the face in 195 cases (25.8%), the cervical region in 116 (15.3%), and the trunk in 96 (12.6%), as described in table 2. Some patients presented more than one affected area.

**Table 2:** Distribution of 232 patients according to the location of the injuries.

Location	Number of patients	%
Face	195	25.8
Cervical area	116	15.3
Trunk (sternum, breasts, scapula)	96	12.6
Upper limbs	75	9.9
Hands	60	7.9
Lower limbs	49	6.4
Scalp	44	5.8
Abdomen/flanks	44	5.8
Feet	27	3.5
Armpits	24	3.1
Lumbar region	11	1.4
Inguinal/genital region	10	1.3
Buttocks	9	1.2
Total	760^a^	100.0

a Some patients had more than one affected region.

Among the 232 patients with ACD to cosmetics, 82 (35.3%) were diagnosed by the cosmetic panel, 65 (28%) by the association of standard panel and extra substances, 55 (23.7%) by the association between cosmetic and standard panel, and 30 (12.9%) by the standard panel.

The main allergens with positive results in the patch tests were toluene-sulfonamide-formaldehyde resin in 69 cases (29.7%), paraphenylenediamine in 54 (26.3%), Kathon CG in 41 (20.7%), and fragrance-mix 1 in 29 (16.4%), as shown in table 3.

**Table 3:** Distribution of positive patch tests among the 232 patients diagnosed with ACD to cosmetics.

Positive substance	%	Sex		Positive substance	%	Sex	
		F	M			F	M
R-TSF^a^	29.7	69	0	Hydroquinone	2.1	5	0
Paraphenylenediamine	26.3	54	7	Triethanolamine	2.1	4	1
Kathon CG®^b^	20.7	41	7	BHT^d^	1.7	4	0
Fragrance-mix 1	16.4	29	9	Lanolin	1.7	4	0
Formaldehyde	8.2	19	0	Bronopol^e^	1.7	4	0
Colophony	7.7	15	3	Chloroacetamide	1.7	4	0
Balsam of Peru	6.0	11	3	Amerchol L-101	1.3	2	1
Parabens	3.4	6	2	Propylene glycol	1.3	3	0
Irgasan^c^	3.0	6	1	Sorbic acid	0.9	2	0
Ammonium thioglycolate	3.0	7	0	ImU^f^	0.9	2	0
Quaternium-15	2.6	6	0	Chlorhexidine	0.4	1	0

Some patients had more than one positive substance on the patch test.

a Toluene sulfonamide - formaldehyde resin.

b Methylisothiazolinone + methylchloroisothiazolinone.

c Triclosan.

d Butylhydroxytoluene.

e 2-bromo-2-nitropropane-1,3-diol.

f Imidazolidinyl urea.

In 154 (66.4%) of the 232 patients with a confirmed diagnosis of allergic contact dermatitis by cosmetics it was possible to specify the cosmetic product responsible for the lesions, as shown in table 4.

**Table 4:** Cosmetic identified as responsible for ACD in 154 patients.

Cosmetic	%	Sex	
		F	M
Nail polish	32.3	75	−
Hair dye	17.2	36	4
Fragrances/perfumes	7.3	15	2
Shampoo and hair products	6.0	12	2
Moisturizing cream	4.7	7	4
Deodorant	4.3	7	3
Sunscreen	1.3	3	−
Soap	0.9	1	1
Hair straightening treatment	0.4	1	−

In some patients, more than one cosmetic was implicated in the etiology of ACD.

## Discussion

Cosmetics are products used daily by a large portion of the population, making them frequent causes of contact dermatitis, both in irritative and allergic forms.

The frequency of ACD to cosmetics in the service in the assessed period was 16.5% among patients who underwent patch tests. These rates are equivalent to the data of the groups that study this subject, ranging from 1% to 17.8% of all ACD cases. These figures depend on the location of the study, the frequency of use of cosmetics in the evaluated population, the availability of medical services and, mainly, access to patch tests and allergens that cause dermatitis. The data presented here reflect the frequency of such a diagnosis in a university service with access to patch tests.^4^

Regarding gender, the study included 208 women (89.7%) and 24 men (10.3%). This fact is in accordance with literature data, corroborating the idea that the greater number of cosmetics used by women favors the greater frequency of sensitization, although the use of these products has increased among men.^4^, ^5^

The time of evolution of the dermatosis until the arrival at the service was 44 months, on average, which reflects the poor access of the public service population to patch tests, delaying the diagnosis and implying greater morbidity. Other studies show mean intervals of 23 and 29 months.^5^

The two regions of the body most affected were the face and neck, coinciding with the areas of greatest contact with cosmetics, both by direct application and by indirect contact.^2^

ACD to cosmetics can be caused by different components of the formulations, such as active ingredients, preservatives, fragrances, emulsifiers, and vehicle components.

Among the active principles, toluene-sulfonamide-formaldehyde resin (R-TSF) was the most common among all allergens surveyed in the sample (29.7%). These cases were exclusively diagnosed among women. This resin is present in nail enamels, allowing for durability after application. It is an allergen still prevalent in Brazil, although fewer cases have been observed in the present service.

Paraphenylenediamine (PPDA), present in permanent and semi-permanent hair dyes, was the second most frequent among women and men. Although the scalp is thick, this allergen is capable of causing reactions that are often intense and severe. Lesions can also occur on the face, eyebrows, neck, and ears, without affecting the scalp. In addition, this allergen is commonly included in impure henna tattoos, causing sensitization in children.^4^

Ammonium thioglycolate, a reducing agent used in hair straightening and perming products, is widely used in Brazil, especially in procedures performed in the domestic environment. It accounted for 3% of cases of ACD to cosmetics. It is an uncommon allergen, but in 2017 a Japanese study showed 4.8% of patients with reactions to products applied to hair that were positive to this agent.^6^

Preservatives had a significant number of reactions, and isothiazolinones were the most frequent. Kathon CG® (methylisothiazolinone + methylchloroisothiazolinone) accounted for 20.7% of positive tests. It is a common preservative in water-based products, such as creams, lotions, shampoos, and baby wipes. It can also be found in products for industrial use, such as wall paints, cutting oils, glues, textiles, and leather. Contact dermatitis induced by this preservative mainly affects the face and hands in adults. In children, the most affected regions are the perioral and genital regions, and the buttocks, due to their presence in wet wipes.^7^

Unpublished data from this service indicate a 17% frequency of sensitization to Kathon CG® in the general population. This frequency was higher in the present study, as it included a specific group of ACD by cosmetics (in which this allergen is more common). The frequency of sensitization increased substantially from 2011 onwards in the United States and Europe; in Brazil, Scherrer and Rocha demonstrated an increase in sensitization from 3.35% to 11.14% between 2006 and 2012.^8^

Formaldehyde is a well-known antiseptic used as a preservative in cleaning products, cosmetics (*e.g*., shampoos and soaps) and topical medicines. Due to their allergenic and carcinogenic potential, formaldehyde-releasing preservatives can be used instead. In the current study, 8.2% of the cases presented sensitization to formaldehyde, some related to the use of hair straightening products. Although prohibited by the ANVISA as a straightener, it is still used in informal settings, at unknown concentrations. The frequencies of sensitization to formaldehyde range from 2% to 3% in Europe and from 8% to 9% in the United States; therefore, the frequency observed in the present study is similar to the one observed in the United States. The frequency of sensitization to formaldehyde-releasing agents such as quaternium-15 (2.6%), bronopol (1.7%), and imidazolidinyl urea (0.9%) was low.

Paraben sensitization was observed in 3.4% of patients and, although it has become a center of controversy among cosmetic users, it has been the safest and cheapest preservative agent on the market since 1924. It is present in most cosmetic products, toothpastes, mouthwashes, cleaning products, and food. The frequency of sensitization ranges from 0.6% to 2.3% in the studies of the North American group.^9^

Irgasan (triclosan) accounted for 3% of positive tests, a higher frequency than that of the literature (below 1%). This product is used as a preservative in personal care and sports products, bedding, and toys.^10^

Other preservatives, such as BHT (antioxidant), chloroacetamide, sorbic acid, and chlorhexidine, presented low frequencies, and were considered uncommon causes of ACD.

Fragrances are among the most common allergens in cosmetics and, in this study, accounted for 16.4% of positive tests. This value was lower than that observed in the literature, where these substances account for 30% to 40% of cases of allergy to cosmetics. Fragrance-mix 1, balsam of Peru, and colophony are considered as markers of ACD to fragrance in Brazil.

Among the mixes, only the fragrance-mix 1 (cinnamic alcohol, cinnamic aldehyde, hydroxycitronellal, amylcinnamaldehyde, geraniol, eugenol, isoeugenol, and oakmoss absolute) was analyzed in the current study, a fact that could reduce the diagnosis of ACD by fragrances by 15% to 33% when compared with foreign studies, in which fragrance-mix 2 is routinely used in patch tests.^11^

Balsam of Peru is a natural resin composed of more than 250 different chemical substances and is used as a fragrance fixator. In the present study, the frequency of sensitization to this substance was 6% and was related to cases of sensitization to fragrances in six cases (43%), while the other eight had isolated positivity. It is estimated that at least 50% of cases of allergy to fragrances have positive reactions to balsam of Peru, as observed in the present study.^2^

Colophony is a plant-based resin composed of a complex mixture of acidic (90%) and neutral (10%) resins. The abietic and dehydroabietic acids are the most important, and the allergens are products of their oxidation. This resin has multiple uses; its use in perfumes is uncertain, but cross-reactions between substances are well described. In the studied group, 18 patients (7.7%) were sensitive to it, and in 17 cases (7.3%) there was concurrent reaction with fragrance-mix 1. A study by the same group that studied fragrance sensitization, published in 2018, showed 10% of concurrent positive tests.^12^

The products used as vehicles in cosmetics were responsible for nine cases of ACD (2.8%). Among these, lanolin (1.7%) and Amerchol L-101 (1.3%) are noteworthy. The former is extracted from sheep’s wool and widely used in cosmetics and topical medicines. The latter is a commercial product that contains lanolin alcohols obtained from its hydrolysis. Although both are frequently implicated in the ACD of patients with ulcers of the lower limbs and, more recently in patients with atopic dermatitis, they also appear as allergens to cosmetics, as demonstrated in the present study.^13^ In cosmetics they can be present in deodorants, eye cosmetics, depilatories, lipsticks, lotions, and moisturizing creams, in addition to shampoos and conditioners.

Propylene glycol is a synthetic alcohol that can be used as an emollient, solvent, preservative and emulsifier, being found in various products such as cosmetics, personal care products, medicines (including corticosteroids), food, and recently in electronic cigarettes. In this study, test positivity was observed in 1.3% of patients; this finding is in agreement with the literature, where the frequency varies between 0.8% and 3.5% of cases.^14^

Another positive allergen in the tests was triethanolamine, an emulsifier used in cosmetics and topical medications, positive in 2.1% of cases, a value below that observed by Silva et al. in a 2012 publication.^15^ In that work, the authors observed 9.52% positivity for this substance. Results may vary between different locations due to the use of different cosmetics and even due to patients’ access to patch tests.

In the present study, it was possible to identify the products that were responsible for the condition in 66.4% of patients diagnosed with ACD to cosmetics (Table 4). In these cases, the current relevance of the patch tests performed was probable or certain. Probable relevance was defined as tests that were positive for allergens (and sometimes also for the patient's own cosmetics) and there was proof of the composition of the products in use (compatible sources of exposure); the relevance was considered certain when, in addition to the criteria mentioned, there was a recurrence of the condition after re-exposure to the product that caused the condition. In the remaining 33.6%, relevance was considered possible, because despite positive tests for cosmetic allergens and the history of exposure to sources that are known to contain them, the exact composition of the products in use was not available.

Nail polishes were the most common, followed by hair dyes, perfumes, shampoos and other hair products, body moisturizers, deodorants, sunscreens, soaps, and hair straightening products, popularly known as progressive hair brush. The culprit cosmetics vary according to consumption habits, hygiene habits, and cultural and religious traditions. A study carried out in India with patients presenting ACD to cosmetics has indicated face creams, hair dyes, and soaps as the most common. In Brazil, painting nails is a common habit among women, making enamels common ACD agents.^5^ It is worth mentioning that in cases that were positive for R-TSF in enamels and PPDA in hair dyes, it is easier to establish the probable or certain diagnosis, since these are specific allergens of these products. In turn, in cases that were positive to fragrances and preservatives, the diagnosis is often possible, and the specific cosmetics that cause the condition are not so easily identifiable, as the possible sources of exposure are multiple.

A limitation of the present study was the inclusion of only allergens from standard Brazilian and cosmetic panels, although it is known that there are still other sensitizers frequently found in cosmetics. Currently, there are extended panels, which allow testing of a wider range of substances (such as fragrance-mix 2, cocoamidopropylbetaine, and methyldibromo glutaronitrile, among others); however, as they were not available in this service during most of the analysis period, they were not included in the study.

## Conclusions

The frequency of ACD for cosmetics was 16.5% among patients who underwent patch tests. The analyzed population was predominantly female, whose age ranged from 31 to 60 years, with involvement of the face and cervical region; these findings are in agreement with the literature data. The main allergens involved were toluene-sulfonamide-formaldehyde resin, paraphenylenediamine, Kathon CG®, and fragrance-mix 1. However, the study had some limitations, such as the absence of some allergens for analysis, which are currently considered important worldwide as causing ACD, and whose access is still restricted in this setting. Even in the absence of these allergens, it was possible to draw a profile of the cosmetics that cause ACD in a sample of the Brazilian population.

## Financial support

None declared.

## Authors’ contributions

Mariana de Figueiredo Silva Hafner: Approval of the final version of the manuscript; conception and planning of the study; effective participation in research orientation.

Ana Carolina Rodrigues: Obtaining, analyzing, and interpreting the data.

Rosana Lazzarini: Conception and planning of the study; elaboration and writing of the manuscript; critical review of the literature.

## Conflicts of interest

None declared.
